# Simvastatin promotes NPC1‐mediated free cholesterol efflux from lysosomes through CYP7A1/LXRα signalling pathway in oxLDL‐loaded macrophages

**DOI:** 10.1111/jcmm.12970

**Published:** 2016-09-15

**Authors:** Xiaoyang Xu, Aolin Zhang, Matthew S. Halquist, Xinxu Yuan, Scott C. Henderson, William L. Dewey, Pin‐Lan Li, Ningjun Li, Fan Zhang

**Affiliations:** ^1^ Department of Pharmacology & ToxicologyMedical College of VirginiaVirginia Commonwealth UniversityRichmondVAUSA; ^2^ Department of PhysiologyGuangzhou Medical UniversityGuangzhouChina; ^3^ Department of PharmaceuticsMedical College of VirginiaVirginia Commonwealth UniversityRichmondVAUSA; ^4^ Department of Anatomy & NeurobiologyMedical College of VirginiaVirginia Commonwealth UniversityRichmondVAUSA

**Keywords:** simvastatin, lysosomes, unesterified cholesterol, macrophages, NPC1, LXRα, CYP7A1

## Abstract

Statins, 3‐hydroxyl‐3‐methylglutaryl coenzyme A reductase inhibitors, are the first‐line medications prescribed for the prevention and treatment of coronary artery diseases. The efficacy of statins has been attributed not only to their systemic cholesterol‐lowering actions but also to their pleiotropic effects that are unrelated to cholesterol reduction. These pleiotropic effects have been increasingly recognized as essential in statins therapy. This study was designed to investigate the pleiotropic actions of simvastatin, one of the most commonly prescribed statins, on macrophage cholesterol homeostasis with a focus on lysosomal free cholesterol egression. With simultaneous nile red and filipin staining, analysis of confocal/multi‐photon imaging demonstrated that simvastatin markedly attenuated unesterified (free) cholesterol buildup in macrophages loaded with oxidized low‐density lipoprotein but had little effect in reducing the sizes of cholesteryl ester‐containing lipid droplets; the reduction in free cholesterol was mainly attributed to decreases in lysosome‐compartmentalized cholesterol. Functionally, the egression of free cholesterol from lysosomes attenuated pro‐inflammatory cytokine secretion. It was determined that the reduction of lysosomal free cholesterol buildup by simvastatin was due to the up‐regulation of Niemann‐Pick C1 (NPC1), a lysosomal residing cholesterol transporter. Moreover, the enhanced enzymatic production of 7‐hydroxycholesterol by cytochrome P450 7A1 and the subsequent activation of liver X receptor α underscored the up‐regulation of NPC1. These findings reveal a novel pleiotropic effect of simvastatin in affecting lysosomal cholesterol efflux in macrophages and the associated significance in the treatment of atherosclerosis.

## Introduction

Statins, 3‐hydroxyl‐3‐methylglutaryl coenzyme A reductase inhibitors, are the first‐line medications prescribed for prevention and treatment of coronary artery diseases 1. Statins work by inhibiting hepatic cholesterol synthesis and up‐regulating hepatocyte low‐density lipoprotein (LDL) receptors. These actions promote LDL uptake and reduce blood LDL cholesterol levels (LDL‐C), thereby stemming cardiovascular events. Beyond this cholesterol‐lowering effect, statins have been also found to induce an array of pleiotropic actions on several aspects of atherogenic process, which is unrelated to cholesterol reduction, and these actions are being increasingly recognized as essentials to statins’ efficacy 2, 3, 4, 5, 6. Among these pleiotropic benefits, the stabilization of atherosclerotic plaques and reduction of inflammation could be closely associated with affecting lesional macrophages.

In atherosclerosis, lesional macrophages take up oxidized LDL (oxLDL) and endocytically deliver it to lysosomes, where the oxLDL is hydrolysed to unesterified (free) cholesterol by lysosomal acid lipase. Under normal circumstances, the free cholesterol could be actively transported out of lysosomes—presumably by the Niemann‐Pick C1 (NPC1) 7, 8—and then trafficked either to the endoplasmic reticulum (ER) for esterification and stored as lipid droplets or to the plasma membrane as building blocks, or out of the cell in cases of excess abundance 9, 10. However, when aberrations in cholesterol metabolism and trafficking occur, both cholesteryl ester and free cholesterol could accumulate in the cell. The buildup of cholesteryl ester as lipid droplets renders a foamy macrophage morphology and contributes to the growth of atherosclerotic plaques, and the accumulation of free cholesterol, especially those compartmentalized in lysosomes, has been found to inflict inflammation, one of the major compounding factors in the pathology of atherosclerosis 11. It has been reported that lysosomes filled with free cholesterol are prone to leakage and the release of cathepsins that can elicit cytoplasm deterioration and macrophage necrosis. Moreover, sequestered cholesterol may prevent lysosomes from taking in *de novo* synthesized lysosomal enzymes, causing the proteins to degrade interstitially 12, 13, 14. Inside lysosomes, formed cholesterol crystal precipitates can rupture the lysosomal membrane and subsequently activate inflammasomes and induce inflammatory cytokine secretion 15, 16, 17.

Given that lysosomes are located upstream in oxLDL catabolism and free cholesterol trafficking, the regulation of free cholesterol trafficking out of lysosomes could be a centrally important pleiotropic mechanism by which statins reduce cardiovascular incidents. In this study, we investigated the pleiotropic actions of simvastatin, one of the most commonly prescribed classes of statins, on cholesterol homeostasis in macrophages with a focus on lysosomal free cholesterol egression. We found that simvastatin enhanced NPC1 expression and promoted lysosome‐compartmentalized free cholesterol efflux, which led to reductions in inflammatory factor secretion in oxLDL‐loaded macrophages. The up‐regulation of NPC1 was associated with increased production of 7‐hydroxycholesterol by Cytochrome P450 7A1 (CYP7A1) and subsequent activation of the liver X receptor α (LXRα) signalling pathway.

## Materials and methods

All treatment and analysis reagents and biochemical kits utilized in this study were obtained from commercial sources: Cholesterol quantification kit, simvastatin, progesterone, nile red, filipin and 7β‐Hydroxycholesterol (Sigma‐Aldrich, St. Louis, MO, USA); anti‐NPC1 antibody (EMD Millipore, Billerica, MA, USA); Mouse interleukin (IL)‐1 beta/IL‐1F2 Quantikine ELISA kit (R&D Systems, Minneapolis, MN, USA); GenMute^™^ siRNA Transfection Reagent (SignaGen Laboratories, Gaithersburg, MD, USA); oxLDL (TBARS: 29‐44 nmoles MDA/mg; Alfa Aesar, Ward Hill, MA, USA); rabbit anti‐mouse CD68 antibody (Bioss, Woburn, MA, USA); Alexa fluor 633 goat anti‐rat IgG (Life Technologies, Carlsbad, CA, USA); LXRα siRNA, NPC1 siRNA and LAMP‐1 rat monoclonal antibody (Santa Cruz Biotechnology, Dallas, TX, USA); cytochrome P450 7A1 siRNA (Origene, Rockville, MD, USA); solid‐phase extraction (SPE) column (Silicycle, Quebec, QC, Canada); 22(S)‐hydroxycholesterol (D7) (Avanti Polar Lipids, Alabaster, AL, USA); HPLC column, XTerra RP18, 2.1 × 150 mm, 5 μM (Waters Corporation, Milford, MS, USA); and lysosome enrichment kit (Thermo Scientific, Waltham, MA, USA). C57BL/6J mice were obtained from the Jackson Laboratory (Bar harbor, ME, USA) and cared for under housing and animal protocols approved by the Institutional Animal Care and Use Committee at Virginia Commonwealth University.

### Primary culture of macrophages from mouse bone marrow

Primary culture of mouse bone marrow‐derived macrophages was performed as in our previous studies 18, 19. In brief, femur bones were first dissected from C57BL/6J mice. The bone ends were then removed and the bone marrow in the medullary cavities was flushed out with RPMI media and made into single cell suspensions through gentle up‐and‐down pipetting. Subsequently, the bone marrow cells were collected with two centrifugations and washes and cultured in RPMI‐1640 media supplemented with 15% L‐929 conditional medium, 10% FBS, and 100 I.U./ml penicillin and 100 μg/ml streptomycin at 37°C and 5% CO_2_. After 7 days, these cells were differentiated into macrophages and used for experiments. The identity of these macrophages was confirmed by positive immunostaining for CD68.

### siRNA interference and NPC1 and LXRα expressions

The transfection of NPC1, LXRα or CYP7A1 siRNA into macrophages was performed using GenMute transfection reagents as described previously 19. RNA interference efficiency was confirmed using quantitative real‐time PCR analysis 48 hrs after transfection. The expressions of NPC1 and LXRα after LXRα gene silencing were examined, and the transcriptional levels of LXRα after CYP7A1 gene interference were determined. For the Western blot assay of NPC1, the target bands were visualized and quantified using an Odyssey Imager with the application of infrared fluorescent IRDye 680RD‐conjugated secondary antibodies. The specific primer pairs used for the amplification of NPC1, LXRα and CYP7A1 were 5′‐CATTGAGGTCATCCCATTCC‐3′ and 5‐′CAGCGTTTCCTCCTGAAGAC‐3′, 5′‐CAAGATGCAGGAGACCAGGG‐3′ and 5′‐GCTGACTCCAACCCTATCCC‐3′, and 5′‐AAACCCTCCAGGGAGATGCT‐3′ and 5′‐AGGCATACATCCCTTCCGTG‐3′, respectively. After gene interference of NPC1, LXRα and CYP7A1, additional treatments, like simvastatin and oxLDL, were applied 24 hrs later.

### Simultaneous analysis of lipid droplets and lysosomal free cholesterol with confocal/multi‐photon scanning microscopy

The effects of simvastatin on lysosomal cholesterol homeostasis were determined by simultaneous staining of free cholesterol and lipid droplets using filipin and nile red fluorescent dyes, respectively, as well as by immunolabeling lysosomal marker protein LAMP1. Macrophages in 8‐well chamber slides, after different treatments, were fixed, permeabilized, and labelled with lysosomal LAMP1 and filipin as described in our previous study 19. The cells were then briefly incubated with nile red (0.25 μg/ml) for 10 min. and rinsed in PBS once before mounting coverslips for microscopy imaging. Fluorescence images were taken using a multi‐photon laser scanning microscope (Zeiss LSM 510 NLO META; Carl Zeiss Microscopy, Thornwood, NY, USA) equipped with a 63×/1.4 NA Plan Achromat oil immersion objective lens. Multi‐channel images were collected sequentially to ensure no cross‐talk between channels, and the detector offset and gain settings were maintained for all of the images collected. The fluors imaged were Alexa Fluor 633 (ex 633 nm/em 650–710 nm), filipin (ex 740 nm/em 435–485 nm) and nile red (ex 514 nm/em 535–590 nm). Image Pro 9.1 software (Media Cybernetics, Rockville, MD, USA) was used to quantify the intensities of nile red (lipid droplet) and filipin (free cholesterol) stains as well as analyse the colocalization coefficient (Pearson's) between filipin and lysosomal LAMP1 as described previously 20.

### Lysosome fractionation and cholesterol assay

Lysosomes from macrophages were isolated using a lysosome enrichment kit as in our previous studies 21, 22, 23. In brief, the macrophages were first collected into a lysosome enrichment buffer, broken down using Dounce homogenizer. After removal of cell debris, the cell homogenates were laid on the top of OptiPrep gradients and centrifuged at 145,000 × g at 4°C for 2 hrs, the concentrated lysosome portion was collected and cleansed of OptiPrep with one PBS wash. The total and free cholesterol in the isolated lysosomes were measured using a cholesterol quantification kit.

### Extraction of oxysterols and free cholesterol and LC‐MS/MS assay

Oxysterols in macrophages and free cholesterol from macrophage culture media were extracted using published methods 24. In brief, macrophages from different treatments in 60 mm dishes were first scraped into PBS and lysed through freezing and thawing using liquid nitrogen. A premixed chloroform/methanol (1:1, v:v) solution was subsequently added. After thorough mixing, the oxysterols‐containing organic phase was collected and dried using a nitrogen gas evaporator. The residue was dissolved in a 1 ml ethanol saponification solution containing 0.6 M KOH and 1 mg/ml butylated hydroxytoluene and then hydrolysed at 60°C for 3 hrs. The hydrolysed oxysterols were extracted with chloroform and eluted with isopropanol/hexane (3:7, v:v) through a silica SPE column. The eluate was dried, reconstituted in methanol/water (95:5, v:v), and subjected to LC‐MS/MS. The extraction of free cholesterol from macrophage culture media was conducted in the same manner as that for the extraction of oxysterols from macrophages save that saponification was not used. The identification and quantification of oxysterols were performed using an AB Sciex 4000Qtrap, which utilizes positive electrospray ionization, tandem mass spectrometry, and Analyst^®^ 1.5.2 software (AB Sciex, MA, USA). The sterols were chromatographically resolved using Waters Acquity UPLC and Waters XTerra RP18, 2.1 × 150 mm, 5 μM columns with the application of a mobile phase gradient, created by mixing the mobile phase A (5 mM ammonium acetate in methanol) with B (5 mM ammonium acetate in 15% water and 85% methanol) at a flow rate of 0.250 ml/min.: 100% B from 0 to 2 min., 100% B to 0% from 2 to 15 min., hold at 0% B from 15 to 25 min., and finally re‐equilibration for 2 min., for a total run time of 27 min. The sterols were quantified using a linear equation with 1/x weighting that was derived from parallel‐generated chromatograms (peak area responses) with mixed standards.

### ELISA quantification of IL‐1β secretion from macrophages in culture media

Macrophages were subcultured into 6‐well plates at 5 × 10^5^ cells/well. Seven different treatments were applied to these cells: Control, simvastatin, progesterone+simvastatin, scrambled siRNA, scrambled siRNA+simvastatin, NPC1 siRNA+simvastatin and LXRα siRNA+simvastatin, in combination with oxLDL. After treatments for 24 hrs, the culture media were collected and centrifuged to removal of cell debris. Interleukin‐1β in the media was analysed using an IL‐1β ELISA kit according to the manufacturer's manual and our published study 18. Chemiluminescent absorbance was measured with a microplate reader at λ(nm) = 450 and corrected for background readings at λ(nm) = 570. The quantity of IL‐1β was obtained using the generated standard curve.

### Statistics

Data were presented as mean ± S.E. Significant differences between and within multiple groups were examined using anova. Student's *t*‐tests were used to evaluate the significance in differences between two groups of observations. *P* < 0.05 was considered statistically significant.

## Results

### Simvastatin reduced free cholesterol accumulation in macrophages treated with oxLDL

To investigate simvastatin's pleiotropic effects on lysosomal cholesterol accumulation, we first examined oxLDL loading on free cholesterol buildup in lysosomes. We simultaneously applied the lipid dyes nile red and filipin as well as lysosomal marker labelling LAMP1 to visualize free cholesterol compartmentalized in lysosomes and cholesteryl ester in cytosolic lipid droplets. Confocal images showed that oxLDL at lower concentration loading (0–25 μg/ml) caused limited free cholesterol accumulation, observed as moderate blue staining by filipin. At higher concentrations (25 up to the tested limit of 100 μg/ml), however, oxLDL caused profound free cholesterol accumulation in lysosomes as evidenced by the colocalization of the blue filipin staining with Alexa fluor 633‐labelled LAMP1 (Fig. S1A–C). The oxLDL concentration 40 μg/ml was thus used in this study. The pleiotropic effects of simvastatin on intracellular cholesterol accumulation were examined at 10 μM, a concentration that was commonly used in previous studies 25, 26.

**Figure 1:**
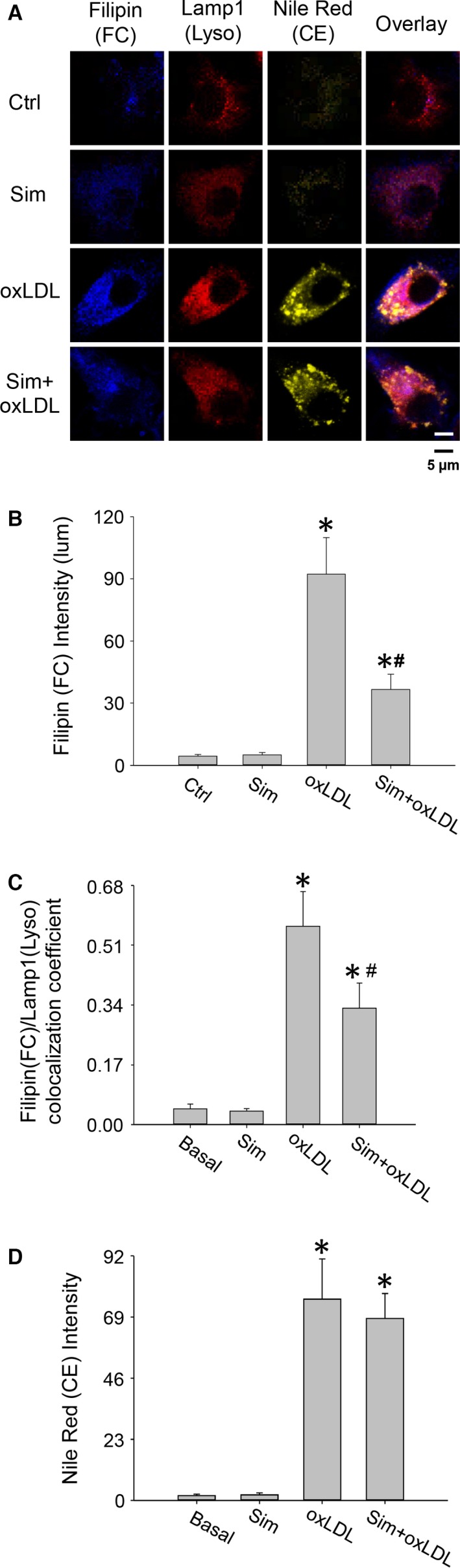
Confocal images showed simvastatin reduced lysosomal free cholesterol in macrophages loaded with oxLDL. Macrophages were incubated with oxLDL (40 μg/ml) in the presence of simvastatin (10 μM) for 48 hrs and labelled with nile red (lipid droplets of cholesteryl ester, yellow), filipin (free cholesterol, blue) and LAMP1 antibody (lysosomes, red). Simvastatin markedly reduced filipin blue but had little effect on yellow droplet intensity in oxLDL‐loaded macrophages. The overlaid images and colocalization coefficient analysis demonstrated that the reduction of filipin staining was mainly from the decrease of lysosomal compartmentalized cholesterol. Simvastatin alone barely had effects on cellular cholesterol levels, compared with control. (**A**) Confocal/multi‐photon microscopy images from nile red, filipin and LAMP1 staining; (**B**) summarized intensities from free cholesterol staining by filipin; (**C**) colocalization coefficient between filipin (free cholesterol) and LAMP1 (lysosomes); and (**D**) summarized intensities from the staining of lipid droplets (cholesteryl ester). *, # *P* < 0.05; * *versus* Ctrl or Sim group, # *versus* oxLDL group, *n* = 6. Sim: simvastatin; FC: free cholesterol; CE: cholesteryl ester; Ctrl: control.

Our confocal imaging results showed that simvastatin significantly reduced lysosomal free cholesterol levels in macrophages loaded with oxLDL, as determined by the intensity of blue filipin staining and colocalization coefficient analysis between filipin and fluor 633‐labelled lysosomal LAMP1 (Fig. 1A–D). However, simvastatin alone exhibited only modest effects on the lipid droplets and lysosomal cholesterol when compared with control.

**Figure 2:**
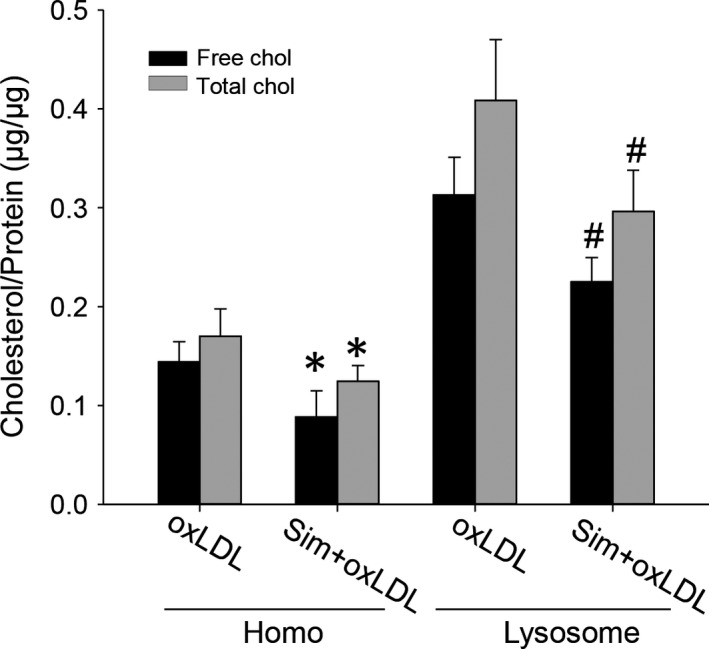
Biochemical measurements showed simvastatin reduced lysosomal cholesterol from oxLDL‐loaded macrophages. Lysosomes were fractionated from macrophages incubated with oxLDL in the presence of simvastatin for 48 hrs by gradient ultracentrifuge; the cholesterol in lysosomal preparations was measured with a kit. Homo: macrophage homogenates, free (Total) chol: Free (Total) cholesterol. *, # *P* < 0.05; * *versus* oxLDL in the Homo group, # *versus* oxLDL in the Lysosome group, *n* = 4.

To further confirm the effects of simvastatin in lowering lysosomal cholesterol, we fractionated lysosomes using gradient ultracentrifugation and directly measured cholesterol contents in the lysosomal preparations. Consistent with confocal imaging results, biochemical analyses showed that free cholesterol constituted a major portion of total cholesterol segregated in lysosomes, and after simvastatin treatment, free cholesterol levels in lysosomes were significantly reduced (Fig. 2).

### Reduction of lysosomal cholesterol accumulation by simvastatin increased cholesterol transport out of macrophages

**Figure 3:**
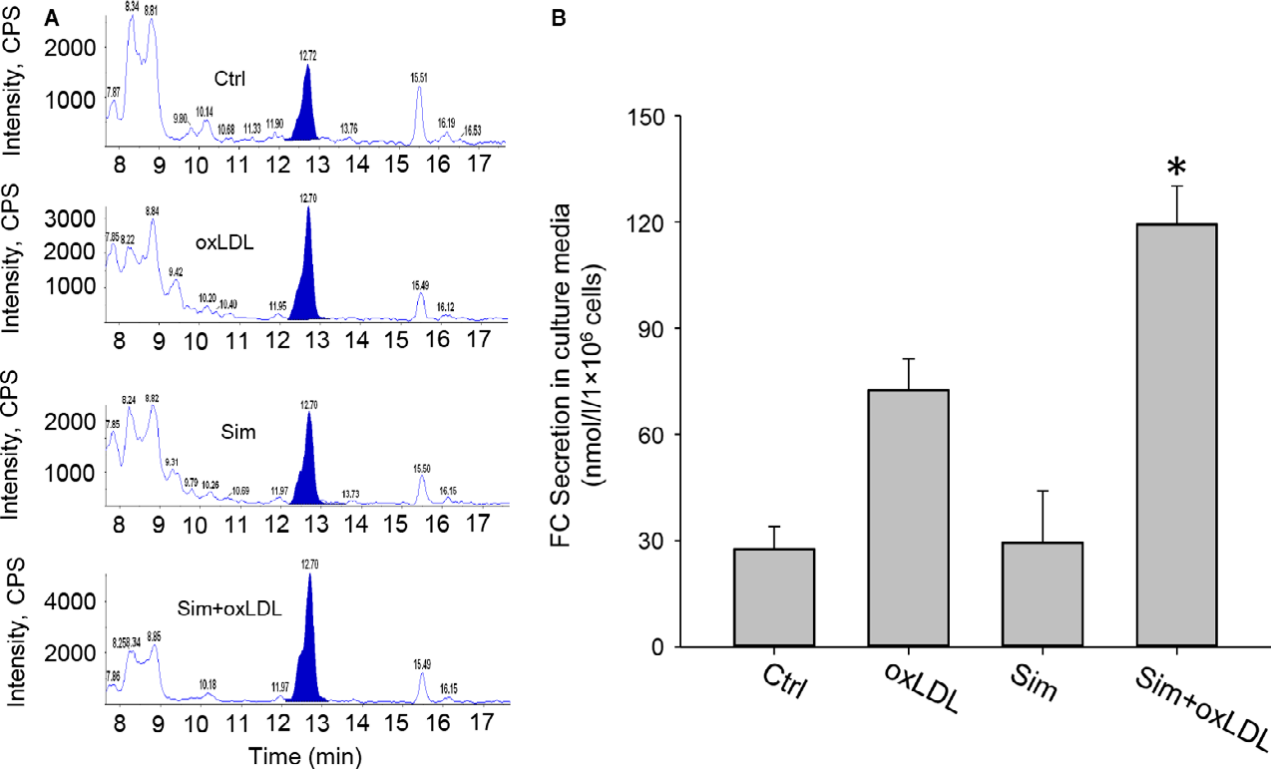
LC‐MS/MS measurement showed simvastatin's reducing lysosomal cholesterol was associated with elevated secretion of free cholesterol from the cells. Macrophages were incubated with oxLDL at the presence of simvastatin for 48 hrs; the culture media were collected to extract cholesterol for LC‐MS/MS assay. (**A**) Representative MS spectrum graphs of free cholesterol, filled peaks, among different groups; (**B**) summarized free cholesterol levels in culture media. *P < 0.05; *versus* any other group, *n* = 3.

It has been reported that LDL endocytosis‐derived free cholesterol in lysosomes has three trafficking destinations: to ER for esterification, to the plasma membrane where it could be transported out of the cell and a small fraction to the Golgi apparatus 27. Since simvastatin reduces lysosomal free cholesterol levels with no significant effects on lipid droplets, it is possible that the reduction in lysosomal cholesterol might be associated with enhanced free cholesterol transport out of cells. We, therefore, proceeded to extract free cholesterol from cell culture media and measure their contents using LC‐MS/MS. As expected, LC‐MS/MS analysis showed that free cholesterol levels in the culture media from the sim+oxLDL group were significantly higher than those in other groups (in nmol/l/10^6^ cells): 119.38 ± 10.75 *versus* 27.69 ± 6.28, 72.49 ± 8.86 and 29.50 ± 14.49 in control, oxLDL and simvastatin only groups, respectively (Fig. 3).

### Simvastatin promoted lysosomal free cholesterol egression through up‐regulation of NPC1 expression *via* LXRα

**Figure 4:**
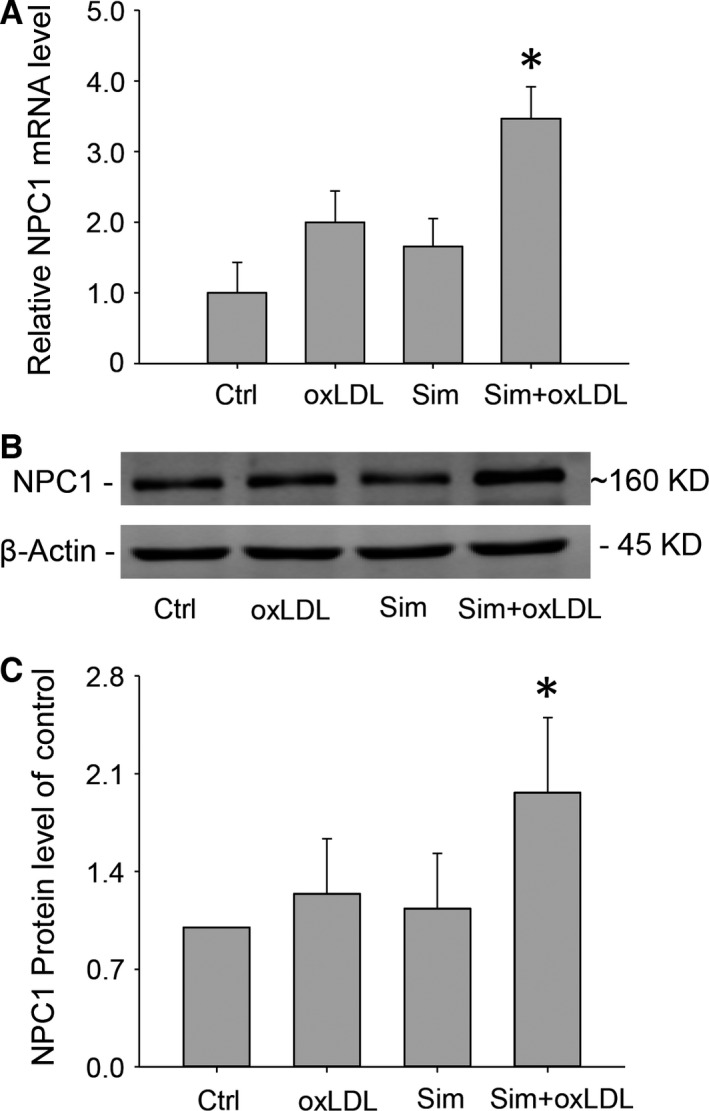
Simvastatin increased NPC1 expression in oxLDL‐loaded macrophages. Total RNA and protein were extracted 24 and 48 hrs, respectively, from macrophages incubated with oxLDL (40 μg/ml) at the presence of simvastatin (10 μM) to determine the expressions of NPC1. (**A**) Summarized NPC1 mRNA levels by quantitative RT‐PCR analysis, (**B**) representative W. B. images of NPC1 bands, and (**C**) summarized intensity from NPC1 bands in W. B., which was normalized to control. *P < 0.05 *versus* any other group, *n* = 6 (**A**), *n* = 5 (**B** and **C**).

**Figure 5:**
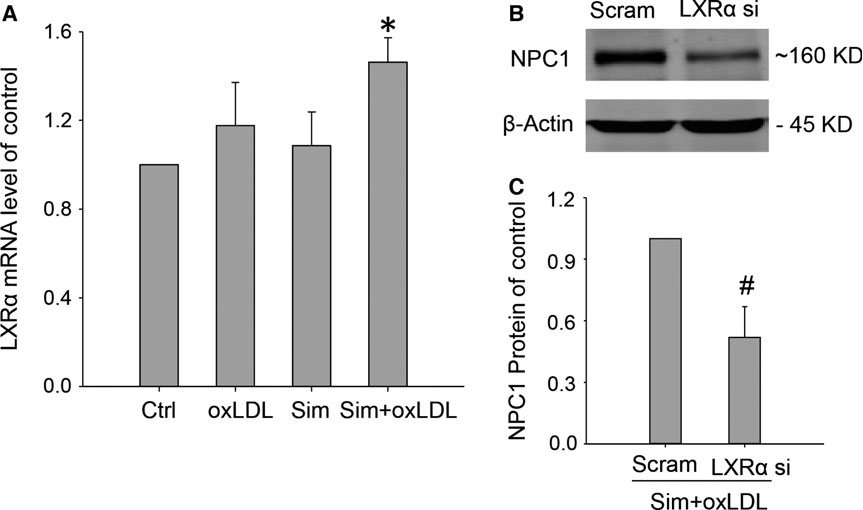
Simvastatin's up‐regulation of NPC1 in oxLDL‐loaded macrophages was mediated by LXRα. (**A**) Quantitative RT‐PCR results of LXRα transcriptional levels in macrophages after 24‐hr incubation with oxLDL (40 μg/ml) and simvastatin (10 μM), (**B**) NPC1 band image from W. B. conducted in oxLDL+simvastatin‐treated macrophages with LXRα gene interference, (**C**) Summarized intensity of NPC1 band from W. B., which was normalized to scrambled RNA group. *, # *P* < 0.05, * *versus* other group, # *versus* Scram; *n* = 5. Scram: scrambled RNA, si: siRNA.

Niemann‐Pick C1 is a free cholesterol transporter residing on late endo/lysosomes that has been found to be responsible for free cholesterol efflux from lysosomes. We, therefore, proceeded to investigate how simvastatin enhanced free cholesterol egression from lysosomes by examining whether simvastatin affected the expression of NPC1. RT‐qPCR and Western blot results demonstrated that in oxLDL‐loaded macrophages, simvastatin elicited significant increases in NPC1 expressions, while oxLDL or simvastatin treatment alone caused only modest changes in NPC1 levels (Fig. 4A–C). While cholesterol is essential for normal cell functioning, excess cholesterol can cause cellular toxicity. Thus, cells have developed exquisite machineries to maintain cholesterol homeostasis. When cholesterol is insufficient in the cell, the nuclear factor Sterol Regulatory Element‐Binding Proteins (SREBPs) 28 will be proteolytically activated to promote cholesterol uptake and *de novo* cholesterol synthesis 29. In the event of cholesterol excess, however, LXRs will be activated to initiate downstream effectors in enhancing free cholesterol efflux 30. To elucidate how NPC1 expression was up‐regulated by simvastatin, we, therefore, sought to examine the functional role of the transcriptional factor LXRα, one of LXR isoforms highly expressed on macrophages 31. RT‐qPCR analysis indicated that LXRα mRNA had an expression pattern similar to that of NPC1 among the groups control, oxLDL, Sim and Sim+oxLDL (Fig. 5A). Furthermore, after the application of LXRα gene interference, the boosting effects of simvastatin on NPC1 expression were abolished (Fig. 5B and C). These results indicated that simvastatin up‐regulated NPC1 expressions in oxLDL‐loaded macrophages *via* LXRα activation.

**Figure 6:**
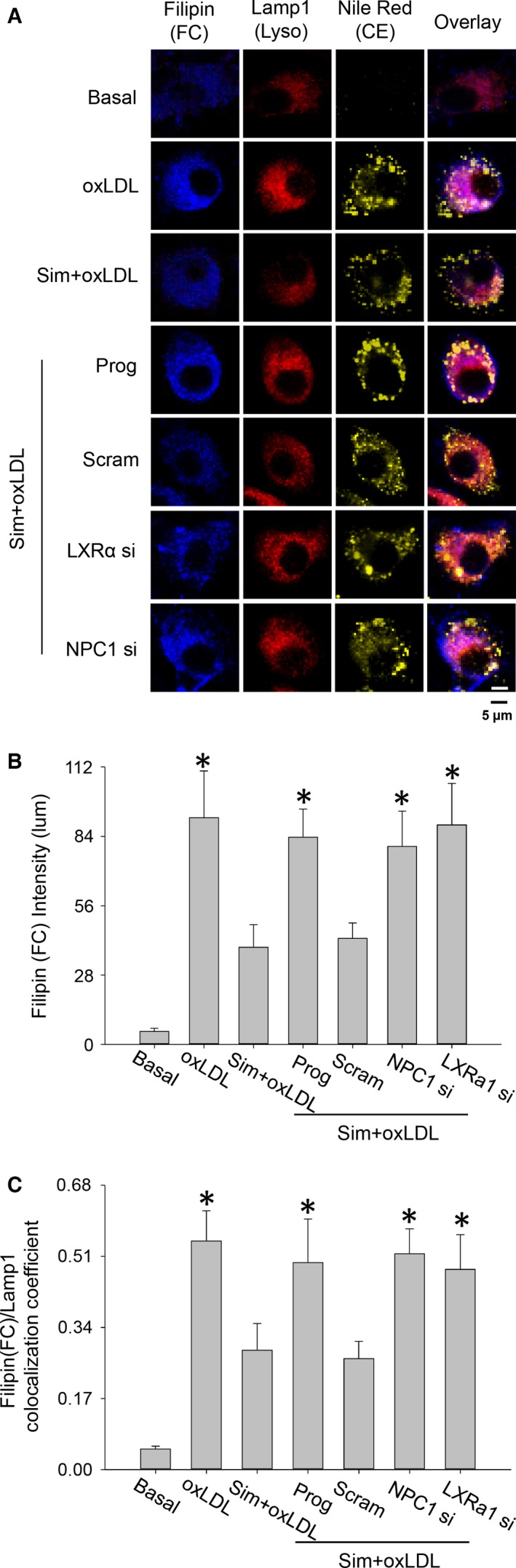
Simvastatin reduced lysosomal free cholesterol was mediated by LXRα signalling. Macrophages were incubated with oxLDL (40 μg/ml) in the presence of simvastatin (10 μM) with NPC1 inhibitor of progesterone or gene interference in LXRα and NPC1 for 48 hrs; the labelling of nile red, filipin and LAMP1 was then performed. (**A**) Confocal fluorescence images showing inhibitions of NPC1 or gene interference of LXRα attenuated simvastatin in its reducing lysosomal free cholesterol, (**B**) summarized intensities from the staining of free cholesterol by filipin, and (**C**) colocalization coefficient between filipin (free cholesterol) and LAMP1 (lysosomes). *P < 0.05, *versus* Sim+oxLDL or Sim+oxLDL plus Scram, *n* = 8. Prog: Progesterone.

Using fluorescence staining and confocal/multi‐photon imaging, we further verified LXRα/NPC1 signalling's involvement in simvastatin‐induced lysosomal free cholesterol efflux. Consistent with simvastatin elevating NPC1 and LXRα expression levels, when NPC1 or LXRα expressions were disrupted by gene interference or when NPC1 was pharmacologically blocked by progesterone (10 μg/ml), simvastatin‐induced lysosomal cholesterol efflux was significantly inhibited as indicated by marked increases in the intensity of the blue from filipin's staining of free cholesterol and an increase in the colocalization coefficient between filipin (blue) and LAMP1 (red) (Fig. 6A–C).

### LXRα signalling cascade in simvastatin‐induced free cholesterol efflux initiated by 7‐hydroxycholesterol

**Figure 7:**
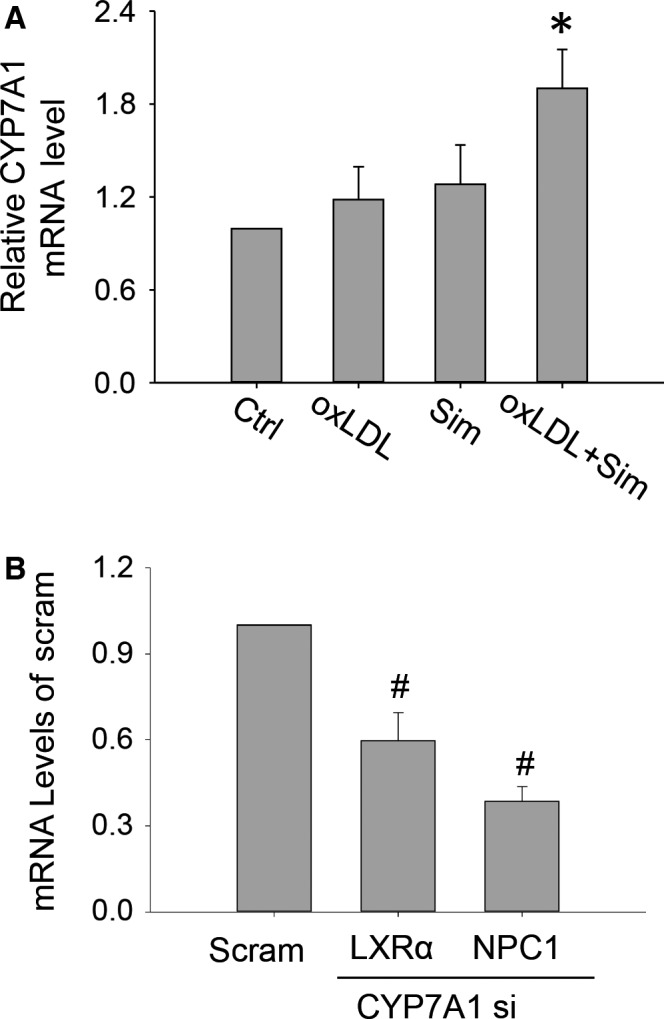
Up‐regulated CYP7A1 expression underscored the elevation of NPC1 and LXRα levels by simvastatin in oxLDL‐loaded macrophages. (**A**) mRNA levels of CYP7A1 in oxLDL+sim‐treated macrophages were markedly increased compared with Sim only, or oxLDL alone group, or control group. (**B**) Gene interference of CYP7A1 significantly decreased LXRα and NPC1 transcriptional levels in oxLDL‐loaded macrophages on simvastatin. *, # *P* < 0.05; * *versus* Ctrl, oxLDL or Sim group; # *versus* Scram, *n* = 3. CYP7A1: Cytochrome P450 7A1.

**Figure 8:**
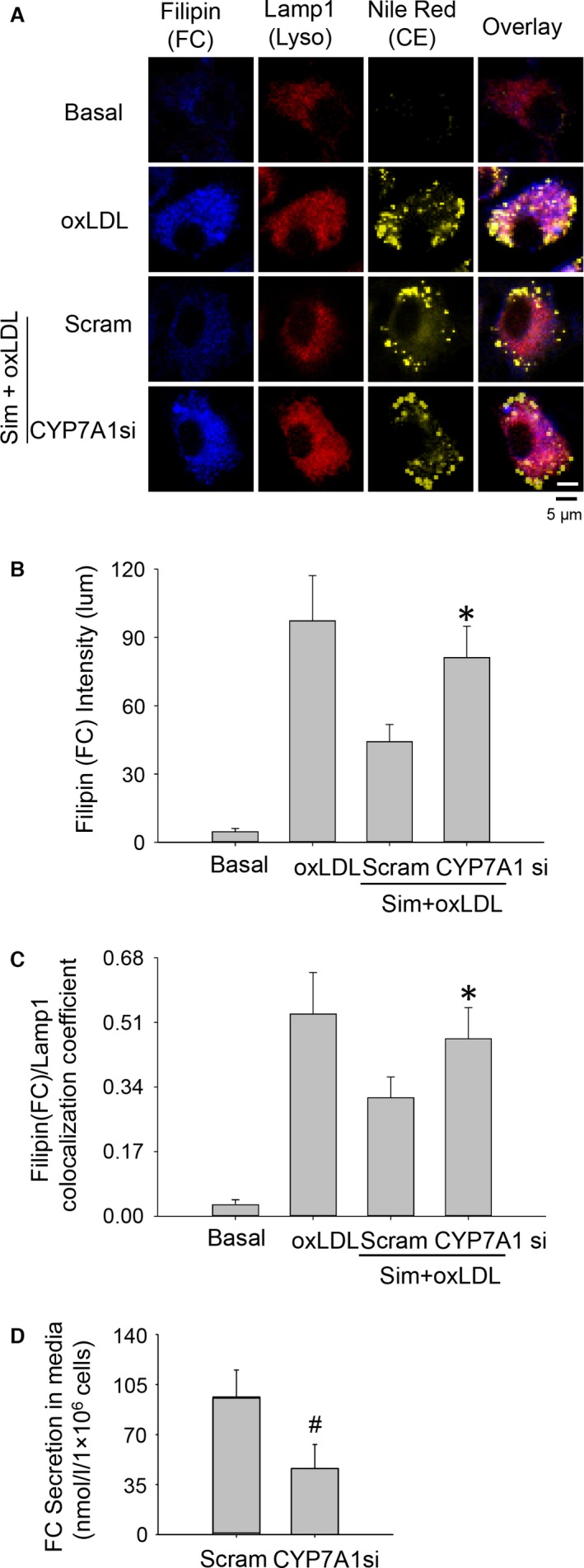
Gene interference of CYP7A1 expression compromised simvastatin‐induced free cholesterol secretion and attenuated the reduction of lysosomal free cholesterol in oxLDL‐loaded macrophages. (**A**) Confocal microscopy images from nile red, filipin and LAMP1 staining; (**B**) summarized intensities from free cholesterol staining; (**C**) colocalization coefficient between filipin (free cholesterol) and LAMP1 (lysosomes), and (**D**) secretion of free cholesterol after CYP7A1 gene interference. *, # *P* < 0.05, * *versus* Scram group; # *versus* Scram group, *n* = 8.

As stated above, LXR signalling serves as a mechanism to promote free cholesterol egression from lysosomes. LXRα is usually activated by elevated production of oxysterols, endogenous LXRα agonists 30. We, therefore, sought to identify the initiator of the LXRα signalling cascade by examining the expression of cytochrome oxidases responsible for generating oxysterols as well as directly measuring intracellular oxysterol levels. Among the major cytochrome P450 oxidases, CH(25)H, CYP27A1, CYP46A1 and CYP7A1, known to endogenously produce oxysterols, we found that the transcriptional levels of CYP7A1 were markedly increased in macrophages treated with simvastatin and oxLDL (Fig. 7A). Consistently, LC‐MS/MS screens showed that levels of cytoplasmic 7‐hydroxycholesterol, the enzymatic product of CYP7A1, were markedly elevated in the sim+oxLDL group compared with the oxLDL‐only group (in pmole/1 × 10^6^ cells) 374.25 ± 52.77 *versus* 287.30 ± 66.94 and that 7‐hydroxycholesterol was not detectable in control and simvastatin groups. When the CYP7A1 gene was disrupted through siRNA interference, the expressions of LXRα and NPC1 were both reduced (Fig. 7B). Correspondingly, fluorescent imaging results showed that, after CYP7A1 gene disruption, simvastatin‐induced lysosomal cholesterol reduction was markedly reduced (Fig. 8A–C); moreover, free cholesterol secretions in culture media of sim+oxLDL‐treated cells were significantly attenuated (Fig. 8D).

### Suppression of pro‐inflammatory cytokine secretion upon reduction of lysosomal free cholesterol accumulation

**Figure 9:**
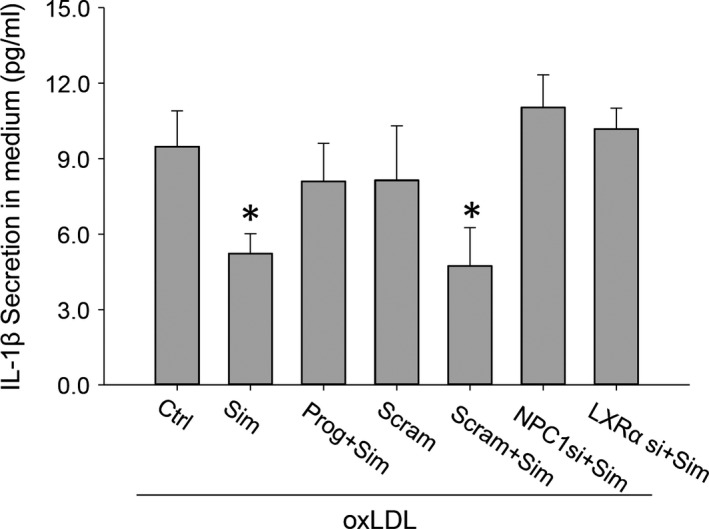
Blocking LXRα/NPC1 signalling pathway functionally attenuated simvastatin‐induced reduction of IL‐1β secretion in oxLDL‐loaded macrophages. Macrophages were loaded with oxLDL while treated with either NPC1 inhibitor of progesterone (10 μg/ml) or gene interference of NPC1 or LXRα; the secretion of IL‐1β in culture media was measured 48 hrs after the treatment. *P < 0.05, *versus* Ctrl, Prog+Sim, Scram, NPC1 si+Sim, or LXRα si+Sim group, *n* = 4.

Accumulated cholesterol in lesional macrophages consists of both free cholesterol and cholesteryl ester. Free cholesterol especially that compartmentalized in lysosomes is cytotoxic and can cause inflammation in atherosclerosis. Therefore, we sought to determine the functional significance of simvastatin‐induced lysosomal free cholesterol egression in the reduction of inflammation. Direct measurements of IL‐1β, a pro‐inflammatory cytokine, revealed that the secretion of IL‐1β in oxLDL‐loaded macrophages was profoundly reduced with simvastatin treatment. Nevertheless, when NPC1 was inhibited by progesterone (10 μg/ml) or disrupted with NPC1 or LXRα siRNA interference, simvastatin‐induced IL‐1β reduction was abolished (Fig. 9).

## Discussion

This study demonstrated that simvastatin up‐regulated NPC1 expression and promoted free cholesterol efflux from lysosomes, which led to reductions in inflammation in oxLDL‐loaded macrophages. The activation of LXRα through elevated production of 7‐hydroxycholesterol by CYP7A1 and consequent up‐regulation of NPC1 expression was the underlying mechanism.

Regarding simvastatin's effects on macrophage cholesterol homeostasis, it has been reported that this cholesterol‐lowering agent prevents cholesterol egression in macrophages not loaded with oxLDL. This effect can be attributed to a feedback response through SREBPs signalling stemming from insufficient levels of intracellular cholesterol owing to simvastatin's inhibition of *de novo* cholesterol synthesis 26, 32. In oxLDL‐unloaded macrophages, we obtained similar results in which simvastatin had only modest effects on free cholesterol efflux. For oxLDL‐loaded macrophages, however, we found that simvastatin markedly promoted lysosomal free cholesterol efflux. Thus, oxLDL loading is a necessary condition for simvastatin to exert its cholesterol‐egression effects on macrophages. In clinical practice, simvastatin is administered to prevent and treat coronary events. Patients with these conditions have often experienced long‐term exposure to hypercholesterolaemia and have been afflicted with advanced atherosclerotic lesions consisting of cholesterol‐accumulated macrophages. Given the similarity of cholesterol accumulation in macrophages *in vitro* and *in vivo*, it is possible that simvastatin could mobilize cholesterol out of lesional macrophages and exert pleiotropic effects therapeutically.

In this study, we showed that simvastatin had different effects on the cholesterol pools in macrophages. While simvastatin profoundly reduced lysosomal free cholesterol accumulation, it exerted little effect in reducing cytosol cholesteryl ester‐containing lipid droplets. This finding is consistent with the clinical observation that statins, including simvastatin, generally display limited capacity in reversing the growth of atherosclerotic plaques 33, of which lipid droplets are a major constituent. Since simvastatin had little effect on lipid droplets overall, the reduction in lysosomal free cholesterol may have been associated with an enhancement in cellular cholesterol efflux. Indeed, LC‐MS/MS measurement demonstrated that simvastatin markedly increased cholesterol secretions in culture media in oxLDL‐loaded macrophages. In this regard, it has been reported that the trafficking of lysosomal free cholesterol to the plasma membrane serves as a major pathway to remove excess cholesterol generated from endocytosed modified LDL 34, 35. The mechanism underlying the transport of lysosomal cholesterol out of the plasma membrane still remains largely unknown. Nonetheless, simvastatin, by promoting free cholesterol egression from lysosomes, markedly reduces inflammatory cytokine production, an indispensable additional benefit that aids in simvastatin's prevention and treatment of atherosclerosis.

Regarding how simvastatin promoted lysosomal cholesterol efflux, we found that enhanced expressions of NPC1 underscored this process. In elucidating the pathway that led to NPC1 up‐regulation, we examined the signalling role of LXRα, a well‐defined nuclear transcription factor that functions to regulate excess cholesterol in cells. By determining the cytoplasmic levels of all major oxysterols, 7‐, 22‐, 24‐, 25‐ and 27‐hydroxycholesterol and desmosterol, known to endogenously activate LXRα, we found that the level of 7‐hydroxycholesterol was significantly increased. As expected, the transcription of CYP7A1, the enzyme responsible for 7‐hydroxycholesterol production, was also markedly elevated. Disruption of CYP7A1 by siRNA gene interference led to the abolition of LXRα‐mediated signalling and its associated free cholesterol egression. In this regard, the role of CYP7A1 in cholesterol homeostasis has been also observed in hepatocytes, in which elevated hepatic CYP7A1 expression and 7‐hydroxycholesterol production promote bile acid formation and cholesterol clearance in hypercholesterolaemia 36. In humans, CYP7A1 deficiency could render a hypercholesterolaemic phenotype and cause cholesterol buildup in the liver 37.

In oxLDL‐loaded macrophages, we found the cellular cholesterol levels were markedly increased but the expressions of LXRα and CYP7A1 only modestly elevated. Given the activation of LXRα signalling as a fundamental machinery protecting cells against excess cholesterol, one would expect a significant up‐regulation in the expressions of LXRα and CYP7A1 with oxLDL loading. It is noteworthy that in our study, the increased free cholesterol in oxLDL‐loaded macrophages is largely confined to the lysosomal compartments, and this lysosomal compartmentalized cholesterol could not be effectively sensed and utilized for oxysterol generation by CYP7A1, the processes known occurred in the ER 38. Therefore, although excess cholesterol has the potential to up‐regulate CYP7A1 expression, its limited availability could eventually compromise the expression of CYP7A1 as observed in this study. Besides up‐regulated by cholesterol, the expression of CYPs also could be pharmacologically induced by many medicines 39. In this regard, it has been found that atorvastatin, one member of statins as simvastatin, acts as an inducer to elevate the expression of CYP7A1 in mice 40. In oxLDL‐loaded macrophages, simvastatin could similarly strengthen the expression of CYP7A1 and boost its enzymatic production of 7‐α‐hydroxycholesterol. The expression of LXRα has been found regulated through an autoregulation mechanism 41, in which the transcriptional activation of LXRα by its ligand oxysterols also leads to the up‐regulation of expression in LXRα itself. Thus, in oxLDL‐only group, without the presence of simvastatin and the subsequent insufficiency in 7‐α‐hydroxycholesterol, the expression of LXRα could be greatly compromised.

In summary, our results for the first time demonstrate that simvastatin promotes free cholesterol efflux from lysosomes and decreases inflammation in oxLDL‐loaded macrophages. These findings will advance our pharmacological knowledge of simvastatin's lesion‐targeted pleiotropic actions in the prevention and treatment of cardiovascular diseases. The findings from this study may also be able to guide us to delineate other satins’ pleiotropic benefits.

## Conflict of interest

The authors confirm that there are no conflicts of interest.

## Author contribution

F. Zhang designed the research study. Xu, A. Zhang, Halquist, Yuan and F. Zhang performed the research. Xu, Yuan and F. Zhang performed data analysis. A. Zhang, Halquist, Henderson, Dewey, P‐L Li, N. Li and F. Zhang wrote or contributed to the writing of the manuscript.

## Supporting information


**Figure S1** Concentration‐dependent effects of oxLDL loading on lysosomal free cholesterol accumulation in macrophages.

**Figure S2** Biochemical measurement of cellular cholesterol extracted from macrophages.

**Data S1** Supplemental methods and materials.
