# Supplementary figures and images for: Simvastatin promotes NPC1‐mediated free cholesterol efflux from lysosomes through CYP7A1/LXRα signalling pathway in oxLDL‐loaded macrophages
Source: J Cell Mol Med. 2016 Sep 15;21(2):364–74. doi: 10.1111/jcmm.12970 (PMC5264135; doi:10.1111/jcmm.12970)

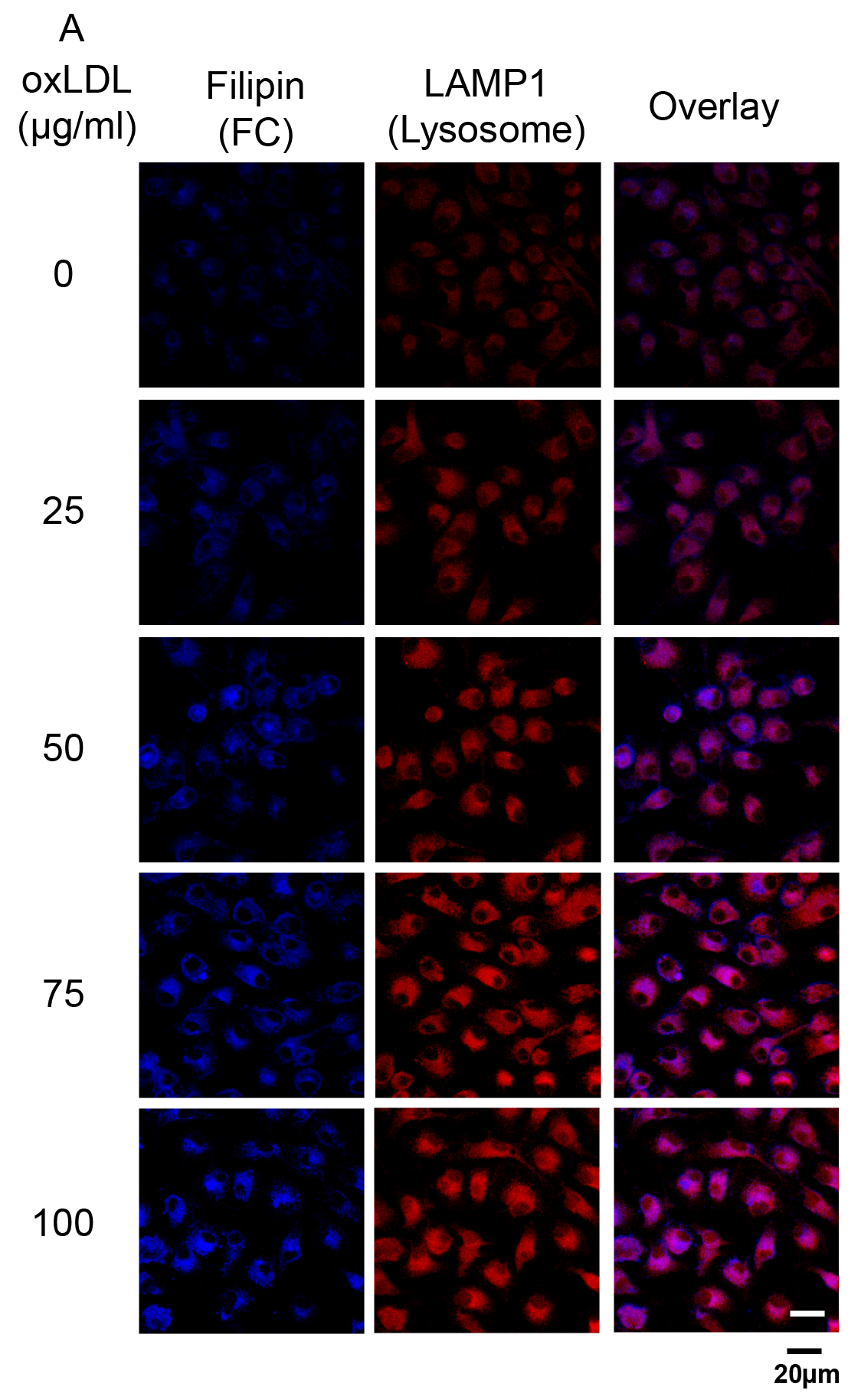

Supplement: Supplementary file 1 — Figure S1 Concentration‐dependent effects of oxLDL loading on lysosomal free cholesterol accumulation in macrophages. [file JCMM-21-364-s001.tif]

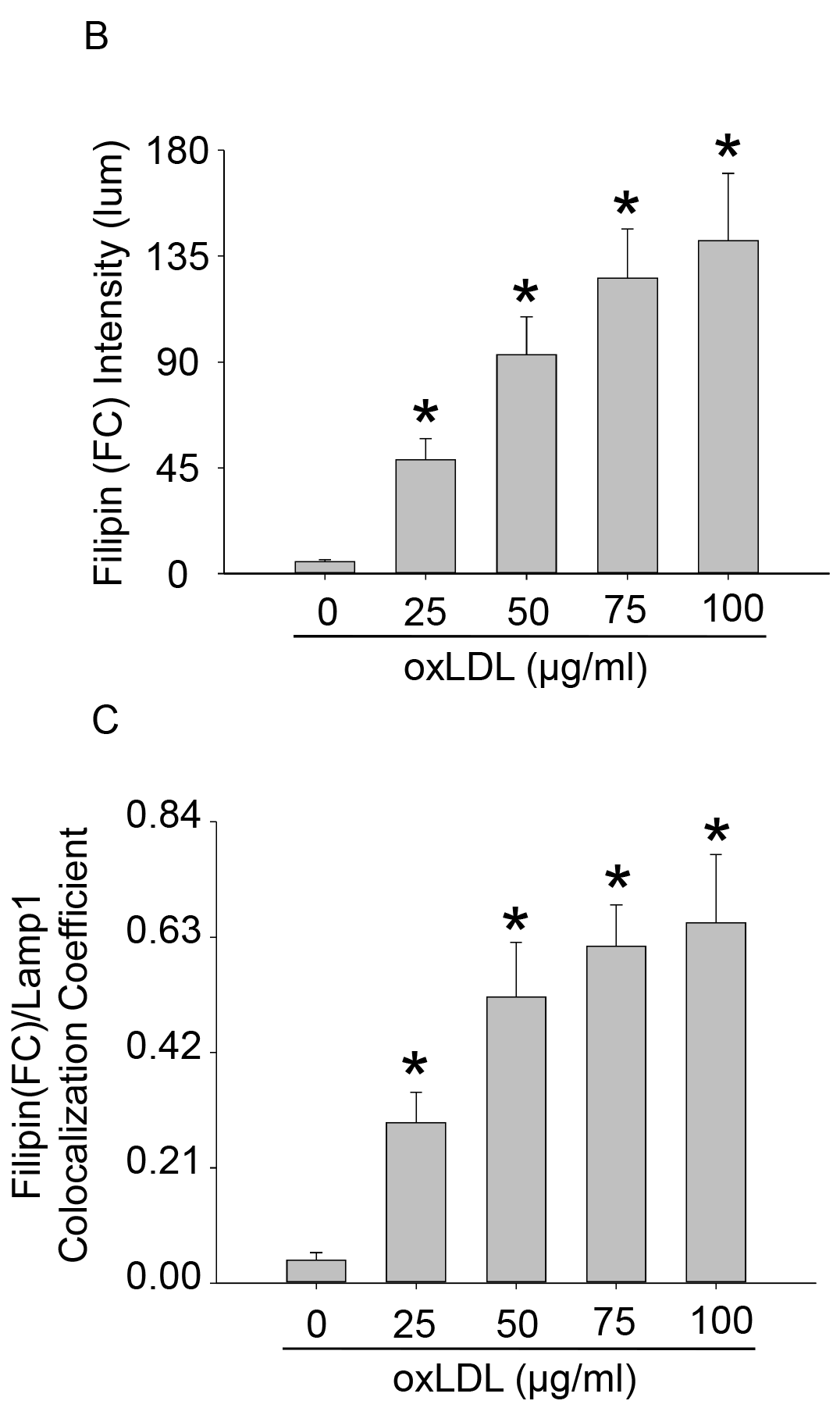

Supplement: Supplementary file 2 [file JCMM-21-364-s002.tif]

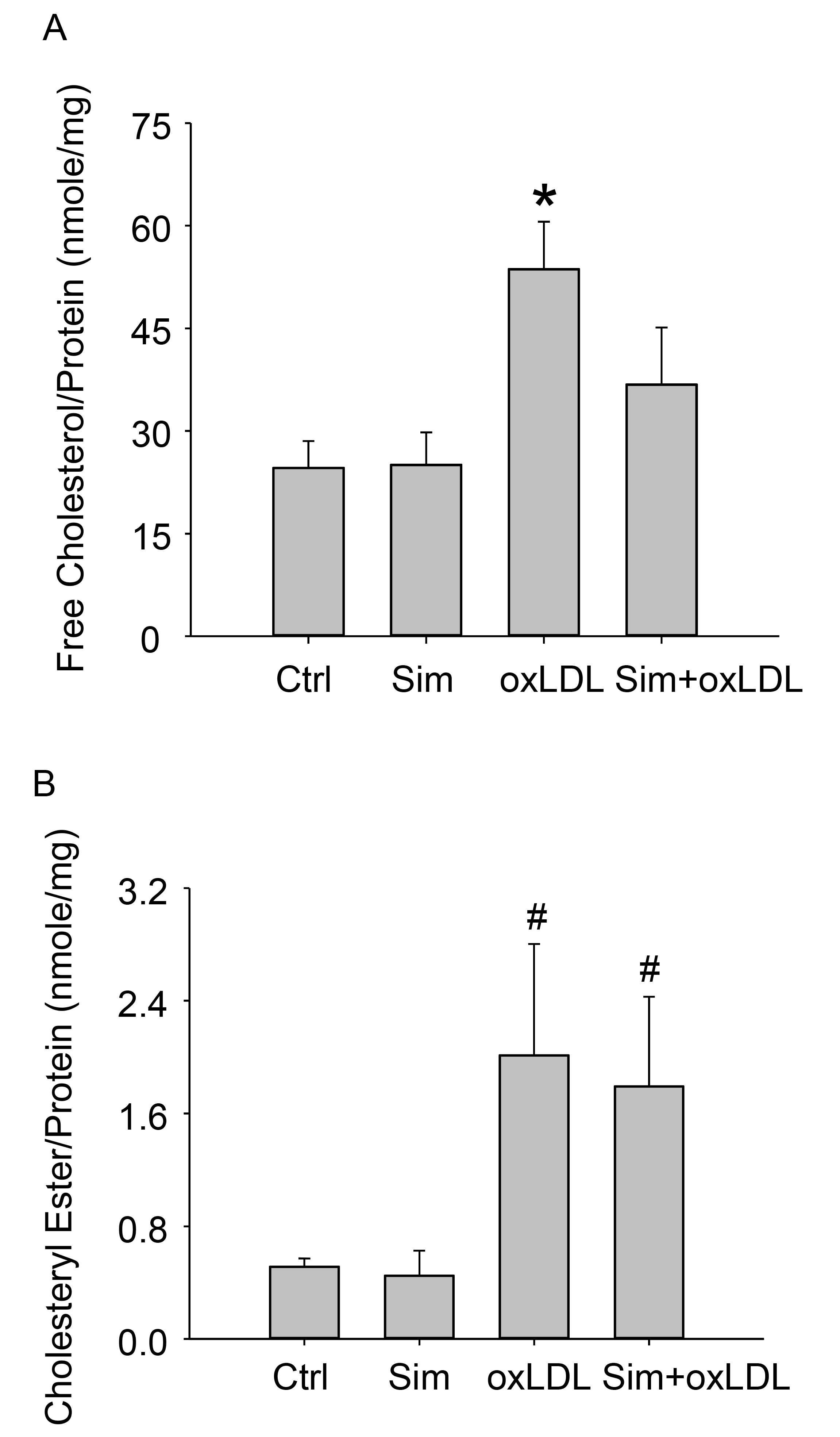

Supplement: Supplementary file 3 — Figure S2 Biochemical measurement of cellular cholesterol extracted from macrophages. [file JCMM-21-364-s003.tif]
