# Supplementary material for: Simvastatin promotes NPC1‐mediated free cholesterol efflux from lysosomes through CYP7A1/LXRα signalling pathway in oxLDL‐loaded macrophages
Source: J Cell Mol Med. 2016 Sep 15;21(2):364–74. doi: 10.1111/jcmm.12970 (PMC5264135; doi:10.1111/jcmm.12970)
Supplement: Supplementary file 4 — Data S1 Supplemental methods and materials. [file JCMM-21-364-s004.docx]

**Supplementary Figure 1 and 2**

**S Figure 1.** Concentration-dependent effects of oxLDL loading on lysosomal free cholesterol accumulation in macrophages. Macrophages were incubated with oxLDL of different concentrations for 48 h and then labeled with filipin (free cholesterol, blue) and LAMP1 (lysosome, red) as described in METHOD section. The accumulation of free cholesterol was determined by measuring blue intensity, and the location of free cholesterol in lysosomes was examined by analyzing the colocalization coefficient between filipin blue and LAMP1 red, using Image Pro 9.1 software (Media Cybernetics, Rockville, MD). A: Confocal microscopy images showed the blue staining of free cholesterol was concentration-dependently increased. B: Summarized blue intensity for the staining of free cholesterol by filipin, C: Summarized colocalization coefficient between filipin blue (Free cholesterol) and LAMP1 red (Lysosomes). **p* < 0.05, comparted with 0 group, n = 5.

**S Figure 2.** Biochemical measurement of cellular cholesterol extracted from macrophages. Macrophages grown in 6-well plates were treated with oxLDL (40 µg/ml) and/or simvastatin (10 µM) for 48 h. The cellular cholesterol were extracted using hexane-isopropanol solvent and measured with Amplex Red Cholesterol Assay kit. The results showed that simvastatin markedly reduced cellular free cholesterol levels in oxLDL-loaded macrophages (A) but had no significant effects on reduction of cholesteryl ester contents compared with oxLDL group (B) *, # p < 0.05; * vs. Ctrl, Sim, or sim + oxLDL group, # vs. Ctrl or Sim group, n = 3. Sim: simvastatin, Ctrl: control.

**Supplemental Methods and Materials**

*Biochemical analysis of free cholesterol and cholesteryl ester levels in macrophages*. The extractions of cellular cholesterol were biochemically determined using Amplex Red Cholesterol Assay kit (Molecular probes, Oregon, USA), the contents of cholesteryl ester were calculated by subtraction of the measured free cholesterol from total cholesterol. Macrophages grown in 6-well plates were treated with oxLDL (40 μg/ml) and/or simvastatin (10 µM) for 48 h. The cholesterol from these cells in groups of control, simvastatin, oxLDL, and simvastatin + oxLDL were extracted with previous methods [1, 2]. In brief, macrophages were collected into PBS and homogenized; hexane-isopropanol (V/V = 3:2) extract solvent was then added to the cell homogenates and thoroughly mixed. The cholesterol-containing organic phase was separated and collected after centrifugation at 14,000 rpm, 4^o^C for 10 min. The solvent was dried under nitrogen gas and the residual of cholesterol was dissolved in cholesterol measurement buffer. The fuorogenic reactions of cholesterol were conducted according to the kit manual, and the fluorometric readings were obtained using a microplate reader at λEx/Em (nm): 540/574. The cholesterol was quantified by referred to the parallel-generated cholesterol standard curve.

**Supplemental References**

[1] J. Folch, I. Ascoli, M. Lees, et al. Preparation of lipide extracts from brain tissue. J Biol Chem. 1951; 191: 833-41.

[2] J. Folch, M. Lees, G.H. Sloane Stanley. A simple method for the isolation and purification of total lipides from animal tissues. J Biol Chem. 1957; 226: 497-509.
